# Adventitial lymphatic capillary expansion impacts on plaque T cell accumulation in atherosclerosis

**DOI:** 10.1038/srep45263

**Published:** 2017-03-28

**Authors:** Timo Rademakers, Emiel P. C. van der Vorst, Isabelle T. M. N. Daissormont, Jeroen J. T. Otten, Kosta Theodorou, Thomas L. Theelen, Marion Gijbels, Andrey Anisimov, Harri Nurmi, Jan H. N. Lindeman, Andreas Schober, Sylvia Heeneman, Kari Alitalo, Erik A. L. Biessen

**Affiliations:** 1 Department of Pathology, Cardiovascular Research Institute Maastricht, Maastricht University, the Netherlands; 2 Institute for Cardiovascular Prevention, Ludwig-Maximilians-University Munich, Munich, Germany; 3 Department of Molecular Genetics, Cardiovascular Research Institute Maastricht, Maastricht University, the Netherlands; 4 Department of Medical Biochemistry, Academic Medical Center, Amsterdam, the Netherlands; 5 Wihuri Research Institute, University of Helsinki, Helsinki, Finland; 6 Departments of Vascular Surgery and Transplantation Surgery, Leiden University Medical Center, Leiden, The Netherlands; 7 Institute for Molecular Cardiovascular Research, RWTH Aachen, Germany

## Abstract

During plaque progression, inflammatory cells progressively accumulate in the adventitia, paralleled by an increased presence of leaky vasa vasorum. We here show that next to vasa vasorum, also the adventitial lymphatic capillary bed is expanding during plaque development in humans and mouse models of atherosclerosis. Furthermore, we investigated the role of lymphatics in atherosclerosis progression. Dissection of plaque draining lymph node and lymphatic vessel in atherosclerotic ApoE^−/−^ mice aggravated plaque formation, which was accompanied by increased intimal and adventitial CD3^+^ T cell numbers. Likewise, inhibition of VEGF-C/D dependent lymphangiogenesis by AAV aided gene transfer of hVEGFR3-Ig fusion protein resulted in CD3^+^ T cell enrichment in plaque intima and adventitia. hVEGFR3-Ig gene transfer did not compromise adventitial lymphatic density, pointing to VEGF-C/D independent lymphangiogenesis. We were able to identify the CXCL12/CXCR4 axis, which has previously been shown to indirectly activate VEGFR3, as a likely pathway, in that its focal silencing attenuated lymphangiogenesis and augmented T cell presence. Taken together, our study not only shows profound, partly CXCL12/CXCR4 mediated, expansion of lymph capillaries in the adventitia of atherosclerotic plaque in humans and mice, but also is the first to attribute an important role of lymphatics in plaque T cell accumulation and development.

Human and experimental murine atherosclerosis are both hallmarked by chronic inflammation. Several immune cell types, including macrophages, T and B cells, mast cells, and dendritic cells (DCs), are thought to be involved in the development and progression of an atherosclerotic plaque^1^,^2^,^3^,^4^,^5^. Although inflammatory responses are largely confined to the intimal plaque itself, there is growing evidence for a crucial role of the adventitia in vascular inflammation and plaque development^6^.

The adventitia is a highly organized tissue harboring stromal cells, vessels and, in particular in atherosclerosis, (resident) leukocyte subsets^7^,^8^,^9^,^10^,^11^,^12^,^13^. The overt presence of DC-T cell clusters in adventitia suggests a role as scaffold for antigen presentation^12^,^14^. Adventitial vessels, the vasa vasorum, expand with atherosclerosis progression both in humans and in mice, and have important functions in inflammatory cell trafficking, amongst others^10^,^15^,^16^,^17^.

The adventitia is therefore increasingly viewed as an important gateway for leukocytes, such as T cells^10^, to the plaque, a notion referred to as the “outside-in” hypothesis^17^. As we have previously shown, adventitial vessels were more leaky than similarly sized vessels in other organs, potentially causing interstitial fluid buildup, the drainage of which requires functional lymphatics^18^. Indeed next to vasa vasorum, the adventitia of human atherosclerotic vessels was recently reported to accommodate an extensive lymphatic capillary network^19^,^20^, at a density that increased with disease severity^19^,^20^. The perivascular lymphatic bed exerts several critical functions, not only in interstitial fluid drainage, but also in reverse cholesterol transport from plaque to liver^21^, and in cell trafficking in and out of tissues^22^,^23^,^24^. This suggests an important, but so far unresolved, contribution of adventitial lymphatics to plaque inflammation and atherosclerosis.

In this study, we have addressed the role of adventitial lymphatic capillaries in a murine model of atherosclerosis by two independent loss-of-function approaches. Our results show augmented T cell accumulation after inhibiting lymph drainage or VEGFR3 dependent lymphangiogenesis, suggesting that the lymphatic capillaries are responsible for T cell drainage from the atherosclerotic lesion. Furthermore, we identified the CXCL12/CXCR4 axis as an important regulator of adventitial lymphangiogenesis.

## Results

### Lymphatic capillaries are present in the adventitia of human and mouse atherosclerotic lesions at densities that increase with plaque progression

Previous work by us and others already showed that the adventitial vasculature (aka vasa vasorum) expands with atherosclerosis progression^15^,^16^,^17^,^18^, and that these vasa vasora are dysfunctional and leaky. Conceivably the enhanced presence of leaky vessels will cause local edema, unless the tissue is properly drained by lymphatics. As lymphangiogenesis and angiogenesis are responsive to partly overlapping cues, this led us to investigate whether adventitial lymphatic vessels expand in response to the atherosclerotic stimulus as well.

**Figure 1:**
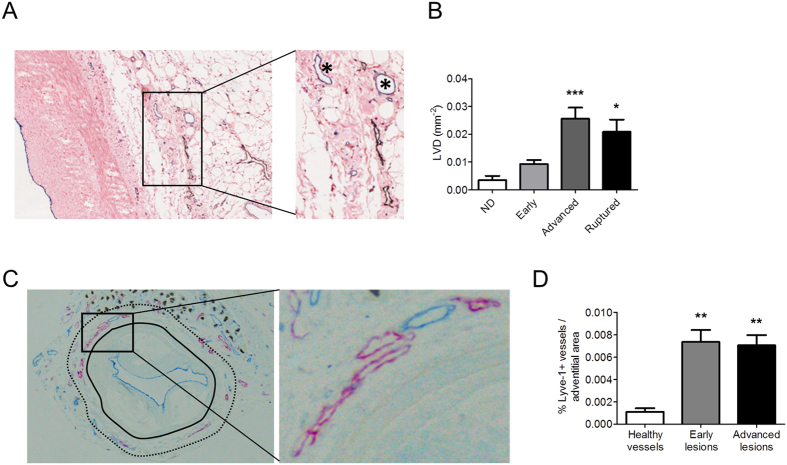
Increased presence of adventitial lymphatic capillaries during human and mouse atherosclerosis development. (**A**) Immunohistochemical staining of podoplanin^+^ (D2-40) lymphatic capillaries (Brown) in human aortic atherosclerotic lesions obtained during kidney transplantation (10x magnification, Insert = 20x magnification). *Shows CD31^+^ adventitial microvessels (blue). (**B**) Quantification of adventitial lymph vessel density (LVD) in different stages of human atherosclerotic lesion development. (**C**) Immunohistochemical staining of Lyve-1^+^ lymphatic capillaries (Purple) and CD31^+^ vasa vasorum (Blue) in mouse carotid atherosclerotic lesions. Continued line indicates the outer border of the media; the area between the continuous and dotted line indicates the adventitial area used for quantification (10x magnification, Insert = 20x magnification). (**D**) Quantification of the percentage Lyve-1^+^ lymphatic capillaries per adventitial area of different stages of mouse lesion development.

Hereto we immunohistochemically screened a cohort of atherosclerotic human aortic artery segments obtained by autopsy, for the presence of adventitial lymphatics. Lymphatic capillaries were abundantly present in the adventitia of human atherosclerotic lesions and their presence was substantially increased in advanced and ruptured lesions compared to non-diseased or early atherosclerotic tissue (Fig. 1A,B). Apparently the pattern of adventitial lymphangiogenesis mirrors that in intimal lesions^19^,^20^. Next we investigated whether lymphangiogenesis also occurs in mouse atherosclerosis. The adventitia of atherosclerotic carotid artery segments from ApoE^−/−^ mice featured both vasa vasorum and lymphatic capillaries (Fig. 1C, red and blue, respectively; Suppl. Fig. 1). The lymphatic capillary density in the adventitia was considerably increased at week 4 and remained high at later stages of plaque development (Fig. 1D), suggesting that lymphatic expansion occurs already at an early stage of atherogenesis.

### Lymph node and lymph vessel dissection deteriorates atherosclerosis development in ApoE^−/−^ mice by promoting T cell accumulation inside the lesion and adventitia

**Figure 2:**
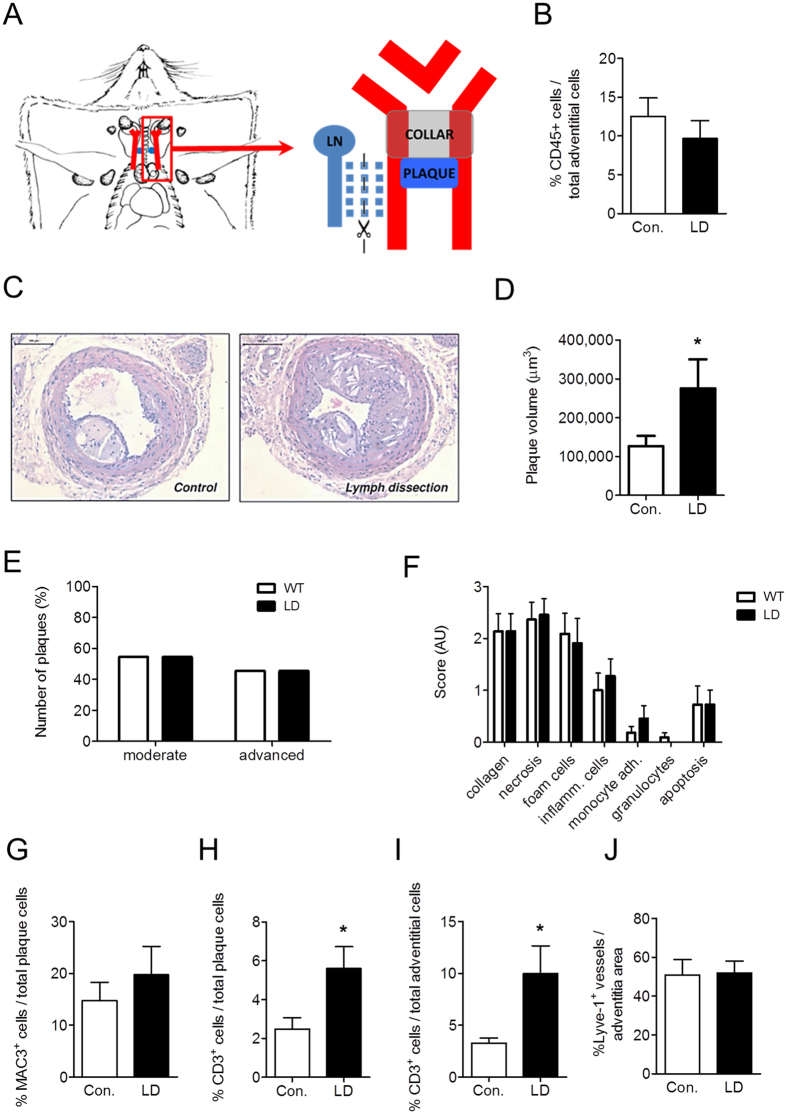
Lymph node dissection results in an aggravation of plaque development in mice, characterized by an accumulation of CD3^+^ T cells within the vessel wall. (**A**) Draining lymph node and efferent vessel were excised in the lymph node dissection group. (**B**) Percentage of CD45^+^ leukocytes in the adventitia of control and lymph node dissected (LD) mice. (**C**) Representative pictures of Hematoxylin-Eosin-stained sections of the carotid arteries from control and lymph node dissected mice. Quantification of (**D**) plaque volume, (**E**) plaque classification and (**F**) routine pathological examination of plaque composition. Percentage of Mac-3^+^ macrophages in the plaques (**G**). Percentage of CD3^+^ T cells in (**H**) plaque and (**I**) adventitia. (**J**) Percentage of Lyve-1^+^ lymphatic capillary area in the adventitia.

The increased presence of lymph vessels in the adventitia of human and mouse atherosclerotic lesions, together with the fact that lymph vessels have been implicated in the regulation of interstitial fluid drainage and inflammation^25^,^26^, argue for an important role for lymph capillaries in atherosclerosis. We therefore studied the impact of dissecting the plaque draining lymph vessels and lymph node on atherosclerosis development in ApoE^−/−^ mice. We opted for the deep cervical lymph node, which is most proximal to the carotid artery bifurcation, and its efferent vessel, running in parallel to the carotid artery (Fig. 2A). Lymph node dissection by itself did neither influence body weight, plasma cholesterol levels (Suppl. Fig. 2A,B) nor induce local inflammation in the adventitia, as judged by the unchanged vascular CD45^+^ leukocyte contents in lymph node dissected mice (Fig. 2B). Lymph node dissection did not affect body weight (29.23 ± 0.48 g and 27.64 ± 0.52 g in control versus lymph node dissected mice, respectively). Interestingly, dissection aggravated atherosclerotic plaque burden (Fig. 2C,D). No significant changes were found in progression stage or gross composition (Fig. 2E,F). Although plaque macrophage content remained unaltered (Fig. 2G), atherosclerotic plaques displayed increased intimal (Fig. 2H) and adventitial CD3^+^ T cell content (Fig. 2I). Circulating monocyte numbers (Suppl. Fig. 2C) and peripheral lymph node T cell content (Suppl. Fig. 2D) were unchanged, again illustrating the local reach of the intervention. Lymph node dissection and thus interruption of drainage did not have any effects on lymphatic capillary bed density in the adventitia (Fig. 2J) at endpoint (4 weeks after lymph node dissection), although origin and functionality of these newly formed capillaries could not be assessed. No differences were observed in vasa vasorum development upon lymph node dissection (Suppl. Fig. 2E). Altogether, these data point to a role of adventitial lymphatic vasculature in drainage of plaque derived T cells and/or of T cell targeting chemokines and cytokines from the plaque, translating in reduced T cell but not macrophage accumulation in the plaque, thus dampening plaque growth.

### Inhibition of lymphangiogenesis using an AAV-hVEGFR3-Ig construct did not change plaque burden, yet increased lesional T cell content

**Figure 3:**
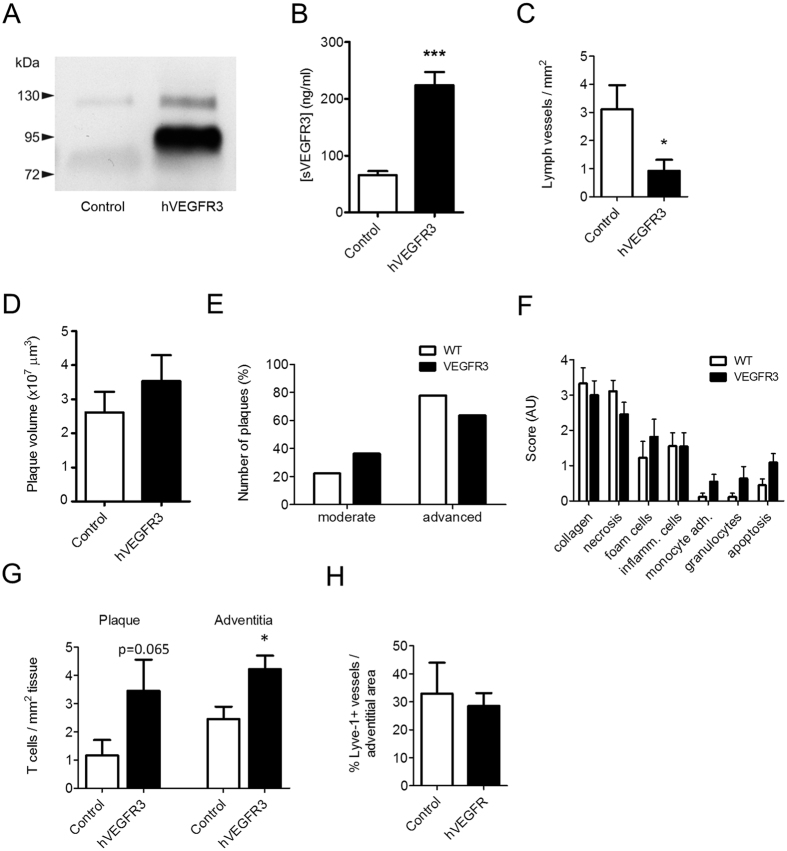
Soluble hVEGFR3 gene therapy did not affect plaque burden and plaque-associated lymphangiogenesis, yet induced an increase in plaque T cell content. (**A**) Western blot showing cropped image of blot and (**B**) ELISA analysis of hVEGFR3 expression in plasma after AAV-hVEGFR3-Ig gene transfer. (**C**) Lymph vessel formation and ingrowth in an *in-vivo* matrigel plug assay, four weeks after s.c. administration of AAV-hVEGFR3-Ig gene transfer. Quantification of (**D**) plaque volume, (**E**) plaque classification and (**F**) routine pathological examination of plaque composition of lesion formation in carotid arteries. (**G**) Quantification of adventitial and plaque T cell content. (**H**) Percentage of Lyve-1^+^ lymphatic capillary area in the adventitia.

Lymph node dissection did not alter atherosclerosis associated adventitial lymph vessel expansion, suggesting that these newly formed lymphatics are not originating from the main efferent lymph node that runs in parallel to the carotid artery. As this potentially points towards a role of lymphangiogenic factors, we next sought to address the contribution of the major driver of postnatal lymphangiogenesis, VEGF-C/D, to adventitial neolymphangiogenesis by AAV aided transfer of IgG-fused soluble human VEGFR3 (AAV-hVEGFR3-Ig)^27^, which inactivates VEGF-C/D. Gene therapy led to persistent overexpression of soluble hVEGR3 in serum of treated but not control mice, as assessed by western blotting (Fig. 3A) and ELISA (Fig. 3B). As expected, the achieved sVEGFR3 levels were sufficient to halt the lymphangiogenic response in an *in vivo* VEGF-C supplemented Matrigel plug assay, as illustrated by the sharp reduction in lymph vessel ingrowth (Fig. 3C). AAV gene therapy did not alter body weight or plasma cholesterol levels (Suppl. Fig. 3A,B), nor did it affect circulating monocyte numbers (Suppl. Fig. 3C) or peripheral lymph node T cell content (Suppl. Fig. 3D). Moreover it did not change plaque volume (Fig. 3D), stage (Fig. 3E) and composition (Fig. 3F). However, plaques of AAV-hVEGFR3-Ig treated mice did show a more inflammatory phenotype, with increased adventitial and intimal CD3^+^ T cell content (Fig. 3G). Much to our surprise, while halting systemic lymphangiogenesis in the matrigel plug assay, hVEGFR3 overexpression failed to inhibit adventitial lymphatic capillary expansion (Fig. 3H). No effects of hVEGFR3-Ig treatment were observed on intimal or adventitial microvessels (Suppl. Fig. 3E). Collectively, in both the systemic lymphangiogenesis intervention study and the LN dissection study, we observed a striking increment in lesional T cell content, pointing to a role of plaque-associated lymph vessels in regulating local T cell content.

### Plaque-associated lymph vessels are partly derived from non-classical lymphangiogenesis via the CXCL12/CXCR4 axis

**Figure 4:**
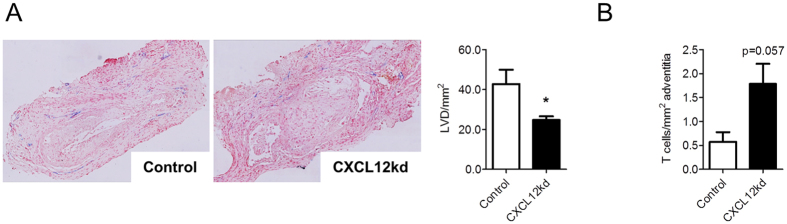
Chemokine and lymph vessel dependent accumulation of T cells. (**A**) Representative Lyve-1 staining and quantification of lymph vessel density (LVD) after perivascular treatment with siRNA targeting CXCL12. (**B**) Representative CD3 staining and quantification of adventitial T cell content after perivascular treatment with siRNA targeting CXCL12.

As observed, surgical interruption of lymph drainage as well as gene therapeutic inhibition of lymphangiogenesis by AAV-hVEGFR3-Ig, both affected plaque T cell accumulation, possibly by attenuating their drainage from plaque (or by reducing their recruitment). However, interfering with the classical VEGF-C/D-VEGFR3 pathway of lymphangiogenesis by using the AAV-hVEGFR3-Ig did not abrogate lymph vessel development at plaque adventitia (Fig. 3H), suggesting a role for non-canonical lymphangiogenic backup factors, that come into play once the VEGF-C/D pathway fails. A chemokine pathway that has been implicated in VEGFR3 independent lymphangiogenesis is the CXCL12/CXCR4 pathway^28^,^29^. This chemokine axis has already been shown to promote lymphangiogenesis in murine models of corneal neovascularization or cancer^28^,^29^. Additionally, CXCL12 has been shown to be a chemoattractant for lymphatic endothelial cells^29^. To address the involvement of CXCR4 signaling in lymph vessel expansion, we have silenced its ligand CXCL12 in adventitia, at plaque initiation, by local perivascular administration of pluronic gel containing siCXCL12. CXCL12 silencing resulted in a significant 42% reduction in adventitial lymph vessel density, underpinning the causal role that CXCR4/CXCL12 plays in atherosclerosis associated lymph vessel expansion (Fig. 4A). In further support of a role of plaque lymphangiogenesis in T-cell accumulation, CD3^+^ T cells were increased after siRNA CXCL12 targeting, at borderline significance (Fig. 4B).

Together, this experiment shows that the lymph angiogenesis is at least partly mediated by CXCL12/CXCR4.

### Human plaque data confirms correlations between lymph vessel density and the CXCR4/CXCL12 axis

**Figure 5:**
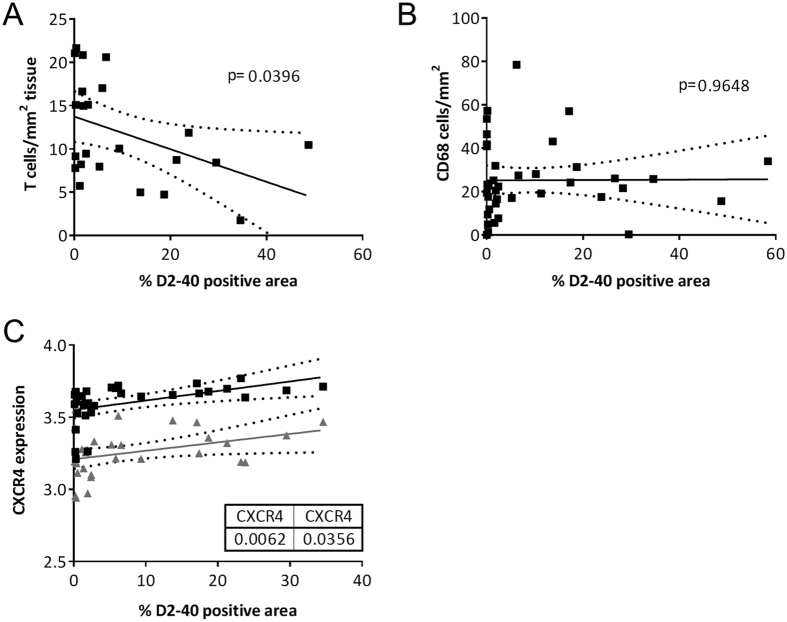
Correlation analysis in human endarterectomy plaque tissues. Correlation analysis between T cells (**A**) and macrophages (**B**) per plaque area and D2-40 positive, lymphatic area in human atherosclerotic lesions. (**C**) Correlation analysis of CXCR4 expression and D2-40 positive, lymphatic area in human endarterectomy plaque tissue.

To confirm a potential role of the local lymphatic vessels in T cell trafficking in humans, we performed correlation analysis between the number of T cells and the local lymph vessel area in human carotid endarterectomy plaque tissue. First, we were able to show a tight correlation between D2-40 staining area in human plaque and mRNA expression of lymphatic markers LYVE-1 and podoplanin (the target of D2-40) (Suppl. Fig. 4). Correlation analysis showed a clear inverse correlation between the amount of T cells and lymph vessel presence in human tissue (Fig. 5A). This correlation seemed to be cell type specific, as macrophages did not show any correlation with the amount of lymph vessel presence (Fig. 5B). As described, we found that the CXCL12/CXCR4 axis at least partly mediates the effects on lymph angiogenesis in mice. Indeed also in human tissue we found a substantial correlation between CXCR4 mRNA expression and lymph vessel presence in human carotid endarterectomy plaque tissue (Fig. 5C), arguing for a link of the CXCR4 axis with plaque lymphangiogenesis.

In conclusion, our data implicate the CXCR4 rather than VEGF-C/D axis in atherosclerosis associated lymphangiogenesis and identified the regulatory role of adventitial lymphatics in plaque T-cell homeostasis.

## Discussion

In this study, we describe a role for adventitial lymphatic capillaries in plaque inflammation and atherosclerosis in mouse and human. As we show, the adventitial lymphatic capillary bed is markedly expanded early on in atherogenesis. Surgical interruption of lymphatic plaque drainage prior to atherogenesis aggravated atherosclerosis development, with increased T cell accumulation in plaque and adventitia as most prominent feature. The latter was also observed after systemic inhibition of VEGFR3-dependent lymphangiogenesis. Interestingly, both interventions did not impact lymph capillary bed density in the adventitia or plaque, suggesting that adventitial lymphatic expansion is not originating from the carotid lymph vessel, and driven by alternative VEGF-C/D independent (backup) mechanisms, possibly through the CXCR4/CXCL12-axis.

As we show, during atherosclerosis, vasa vasorum expansion goes hand in hand with that of the adventitial lymphatic capillary bed. This concurs with previous findings showing progression stage dependent increases in lymphatic capillary content in human carotid artery plaque^19^,^20^, and with the recent observation that lymphatic dysfunction precedes the onset of atherosclerosis^30^. Interestingly, inhibition of VEGFC/D dependent lymphangiogenesis did, in contrast to lymph node/efferent dissection, not affect the atherosclerotic burden, while increasing adventitial and plaque CD3^+^ T cell content. Conceivably, systemic hVEGFR3‐dependent lymphangiogenesis blockage may initially reduce the draining capacity, eliciting alternative VEGFR3 independent lymphangiogenesis pathways in plaque tissue, such as mechano‐induced β1-integrin signaling^31^, or the CXCL12-CXCR4 axis^28^,^29^. In support of the latter Bot *et al*. previously observed elevated CXCR4 expression in atherosclerotic lesions, reflecting an increased lymphangiogenic propensity^32^. Moreover our study shows a positive correlation between CXCR4 expression and lymph vessel presence in human atherosclerotic tissues and reduced adventitial lymph vessel density in mice upon CXCL12 silencing. As such, such VEGF-C/-D independent pathway may also underlie the unchanged adventitial lymph vessel density.

Although CXCR4 appears to be a major cue, in addition to lymphangiogenic growth factors such as VEGF-C and -D^33^, we cannot exclude the involvement of other inflammatory chemokine pathways or other VEGFR3 independent pathways, such as stretch-dependent β1-integrin signaling in adventitial lymphangiogenesis. The different pathways most likely work in concert to induce lymphangiogenesis, with an initial prominent role for hypoxia and inflammation driven VEGF-C/D and CXCL12 production^28^,^29^, followed by an increasing role for stretch-induced β1-integrin signaling upon growth of the plaque and increasing plaque interstitial pressure^31^.

A second relevant question relates to the consequences of adventitial lymphatic bed expansion on plaque inflammation and atherosclerosis. Adventitial lymphatic capillaries could help to prevent edema, caused amongst others by leaky plaque vasa vasorum. In addition, as pointed out by Randolph and coworkers, lymphatics could exert atheroprotective functions by draining plaque contained lipids during regression^21^. Our study adds to this notion in that surgical interruption in their draining function or interference with their expansion aggravated disease. The atheroprotection provided by adventitial lymphatics may be partly based on their regulatory role in plaque T cell accumulation. Whether adventitial lymphatics control CD3^+^ T cell accumulation directly, by mediating egress (as suggested by their close proximity to the adventitial lymph vessels, data not shown), or indirectly by draining T cell chemotactic cues remains to be determined. Dissecting the exact mechanism is complicated by the reciprocal interaction between lymph vessels and T cells with lymph vessels steering T cell trafficking (this study), and T cells in turn inhibiting lymphangiogenesis^34^.

In conclusion, this study shows that adventitial lymphatic vessels increased at an early stage of plaque development, conferring a protective role by draining and therefore dampening the local inflammatory response. Draining lymph node dissection resulted in aggravation of atherosclerosis, accompanied by increased plaque and adventitial T cell content, probably due to interrupted T cell egress or reduced draining of chemotactic cues. Adventitial lymphangiogenesis could not be halted by VEGF-C/D inhibition, but was at least partly arrested due to non-classical lymphangiogenesis via the CXCL12/CXCR4 axis. Further studies are needed to provide further insight into the precise role and mechanisms of adventitial lymphatic capillary formation in atherosclerosis, and their potential impact on clinical outcome.

## Methods

### Immunohistochemistry

Human carotid artery plaque tissue was obtained by endarterectomy, for more detail see ref. 35. For studying adventitial lymph vessels, human aortic plaque tissue was obtained during kidney transplantation, as described previously^36^. Collection, storage and use of tissue and patient data (methods) were performed after informed consent and in agreement with the ‘Code for Proper Secondary Use of Human Tissue in the Netherlands’ and in accordance with the guidelines of, and approved by the medical and ethical committee of Leiden University Medical Center, Leiden, The Netherlands. Lesions were fixed in paraformaldehyde (4%) and paraffin embedded. Histological assessment of human atherosclerotic lesions was performed according to Virmani classification^37^. Lymph vessel analysis was performed using anti-podoplanin (D2-40) antibody (goat polyclonal, R&D systems), and microvessels were stained using CD31 (clone JC/70a, Dako). Vessels were then counted manually and corrected for plaque or adventitial area.

Immunohistochemical (IHC) staining on mouse paraffin carotid artery sections was performed for lymph vessel marker Lyve-1 (rabbit polyclonal, Abcam), for T cells using CD3 (DAKO), for macrophages using Mac-3 (clone M3/74, BD), and for microvessels using CD31 (clone MEC13.3, BD).

Lyve-1^+^ lymphatic capillary content (scored on the basis of morphology and positive staining) was expressed as percentage of adventitial area. Lymphatic capillaries were defined as Lyve-1^+^ cells, circumventing a clearly identifiable lumen. The adventitial area was defined as tissue within a distance of twice the smallest intima-media thickness from the outer elastic lamina. The number of CD3^+^, CXCR3^+^ and Mac-3^+^ cells was expressed as percentage of total plaque or adventitial cells. IHC staining for Lyve-1 was also performed on paraffin embedded matrigel plugs from the AAV-hVEGFR3-Ig study (n = 5/group). Lymph vessel ingrowth into the Matrigel plugs was determined in three slides per plug. All slides were analyzed by a blinded observer (TR) using Leica Qwin software.

### Analysis of adventitial lymphatic capillaries and CD3^+^ T cells

Male ApoE^−/−^ mice (n = 21, 12 weeks of age), backcrossed at least 11 times to C57Bl/6 and obtained from The Jackson Laboratory, were placed on western type diet (WTD) containing 0.25% cholesterol and 15% cacao butter (Special Diets Services, Witham, Essex, UK). Atherosclerotic carotid artery lesions were induced by bilateral placement of semi-constrictive collars^38^. To study adventitial lymphatic capillary density and CD3^+^ T cell content in early (n = 9) and advanced lesions (n = 6), mice were sacrificed 4 and 8 weeks after collar placement, respectively. Naïve carotid artery segments from ApoE^−/−^ mice, not equipped with a perivascular collar (20 weeks of age) served as control (n = 6). All animal experiments were approved by the ethics committee of Maastricht University Medical Center (DEC 2010–098 and 2011–093, Maastricht, The Netherlands) and performed according to institutional guidelines.

### Lymph node and vessel dissection in mice

Male ApoE^−/−^ mice (n = 27, 12 weeks of age) were placed on WTD and semi-constrictive collars were placed around the carotid arteries to induce atherosclerosis development^38^. To address the impact of the deep cervical lymph node drainage to leukocyte influx into and efflux from the adjacent plaque, the lymph node and efferent lymph vessel were removed immediately after collar placement (n = 14). Mice which received only collar placement (n = 13) served as control. At sacrifice, mice from the lymph node dissection group did not show any signs of edema in the neck region. In a separate study we investigated whether lymph node/vessel dissection *per se* induces a local inflammatory response, which could impact lesion formation indirectly. Hereto, we studied effects of the above intervention in normolipidemic wild-type mice (n = 5–6, 12 weeks of age). Both hyper- and normolipidemic mice were sacrificed at 4 weeks after collar placement.

### Pharmacological inhibition of lymphangiogenesis in mice

Male ApoE^−/−^ mice (n = 34, 12 weeks of age) were placed on WTD and semi-constrictive collars were placed bilaterally, proximal to the bifurcation of the carotid artery for shear-dependent atherosclerosis induction^38^. To inhibit VEGFR3-dependent adventitial lymphangiogenesis during plaque development, adeno-associated virus (AAV) encoding a soluble hVEGFR3-Ig (AAV-hVEGFR3-Ig, 10^11^ virus particles per mouse^27^) or control virus (AAV-hVEGFR3(D4-7)-Ig) was administered systemically by intravenous injection at the time of collar placement (n = 17 hVEGFR3-Ig vs. n = 17 control). Mice were sacrificed 4 weeks after collar placement.

A subgroup of mice (n = 5 per group) also received a subcutaneous plug of VEGF-C supplemented Matrigel (BD Bioscience) to validate the efficacy of lymphangiogenesis inhibition by the hVEGFR3-Ig construct. In addition, blood was isolated at 4 weeks after collar placement, serum was obtained by centrifugation (10 min, 1,500 g, 4 °C), and plasma transgene expression was assessed by ELISA and western blot.

### Focal silencing of adventitial CXCL12 expression

Male ApoE^−/−^ mice (12 weeks of age) were placed on WTD (0.15% cholesterol, Altromin, Lage, Germany) for 6 weeks after which semi-constrictive collars were placed around the carotid arteries to induce atherosclerosis. To address the contribution of adventitial CXCL12 to atherosclerosis development, mice were perivascularly treated with a CXCL12 specific siRNA or non-targeting siRNA control (once weekly, 4 nmol, dissolved in plurogenic gel; Dharmacon) as previously described^39^.

### Histology of mouse atherosclerotic lesions

Mice were euthanized by pentobarbital injection (115 mg/kg) and perfused through the left cardiac ventricle with PBS (NaCl/Na_2_HPO_4_/KH_2_PO_4_, pH 7.4) containing sodium nitroprusside (0.1 mg/ml, Sigma) and 1% paraformaldehyde (PFA). The right carotid artery was removed, fixed overnight in 1% PFA and paraffin-embedded sections (4 μm) were cut. To determine plaque volume in the carotid artery, sections were stained for haematoxylin and eosin (HE) and plaque area was measured for consecutive cross-sections, at 100 μm intervals, over a carotid artery segment that covered the entire plaque. Slides were analyzed in a blinded manner using a Leica DM3000 light microscope (Leica Microsystems) coupled to computerized morphometry (Leica Qwin 3.5.1)^40^. Size and progression stage of atherosclerotic lesions was determined as previously described^41^, with minor modifications. Plaques were classified as early (foam cell rich, but lacking a necrotic core), moderately advanced (containing a fibrotic cap and often a necrotic core, but no medial macrophage infiltration) and advanced lesions, typified by medial macrophage infiltrates, elastic lamina degradation and more pronounced necrosis and fibrosis. HE sections were used for morphometric analysis and routine qualitative examination of collagen content, necrosis, foam cell content and amount of inflammatory cells (using scores from 0 (absent) to 3 (high abundance)).

### Flow cytometry and plasma total cholesterol measurement

Blood, spleen and lymph nodes were removed before perfusion (n = 10/group) and used for flow cytometry of monocytes (CD11b^high^ Ly6G^−^, BD), granulocytes (CD11b^high^ Ly6G^high^, BD), T helper cells (CD4^+^, BD), effector T cells (CD8a^+^,BD) and activated T cells (CD44^high^, eBioscience). Briefly, spleen and lymph nodes were homogenized, filtered through a 70 μm mesh. Blood, spleen and lymph nodes were subjected to red blood cell lysis. All cells were stained in flow cytometry buffer (1xPBS, 0.5% BSA, 0.01% NaN_3_). Staining for blood count analyses was conducted using antibodies to CD45, CD115, Gr1, CD19, and CD3 (all eBiosciences) in Hank’s Balanced Salt Solution (HBSS) with 0.3 mM EDTA and 0.1% bovine serum albumin (BSA). Cell counts were estimated utilizing CountBright^TM^ absolute counting beads (Invitrogen). All flow cytometry analysis was performed on a FACS CANTO II (BD Bioscience).

### Statistical analysis

Data are expressed as mean ± SEM. For correlation analyses of human expression data, Shapiro-Wilk test for normality was performed, after which correlations of Gaussian distributed data were calculated by Pearson and of non-Gaussian data by Spearman correlation test. For group comparisons, data were tested for Gaussian distribution, after which a Student t-test (Gaussian) or Mann-Whitney U test (non-Gaussian) was used to compare individual groups; multiple groups were compared by ANOVA or Kruskall-Wallis tests, with Bonferroni or Dunn’s post-hoc test, respectively. Statistics were performed using Graphpad Prism 5.0. A p-value of < 0.05 was considered statistically significant. *Denotes p < 0.05, **denotes p < 0.01, ***denotes p < 0.001.

## Additional Information

**How to cite this article:** Rademakers, T. *et al*. Adventitial lymphatic capillary expansion impacts on plaque T cell accumulation in atherosclerosis. *Sci. Rep.* **7**, 45263; doi: 10.1038/srep45263 (2017).

**Publisher's note:** Springer Nature remains neutral with regard to jurisdictional claims in published maps and institutional affiliations.

## Supplementary Material

Supplementary Information
