# Supplementary material for: Adventitial lymphatic capillary expansion impacts on plaque T cell accumulation in atherosclerosis
Source: Sci Rep. 2017 Mar 28;7:45263. doi: 10.1038/srep45263 (PMC5368662; doi:10.1038/srep45263)
Supplement: Supplementary Information [file srep45263-s1.pdf]

Supplemental Information to manuscript:

Adventitial lymphatic capillary expansion impacts on plaque T cell accumulation in atherosclerosis

Timo Rademakers[*], Emiel P.C. van der Vorst[*], Isabelle T.M.N. Daissormont[*], Jeroen J.T. Otten, Kosta Theodorou, Thomas L. Theelen, Marion Gijbels, Andrey Anisimov, Harri Nurmi, Jan H.N. Lindeman, Andreas Schober, Sylvia Heeneman, Kari Alitalo, Erik A.L. Biessen.

*Shared first authorship

Supplemental Figure 1

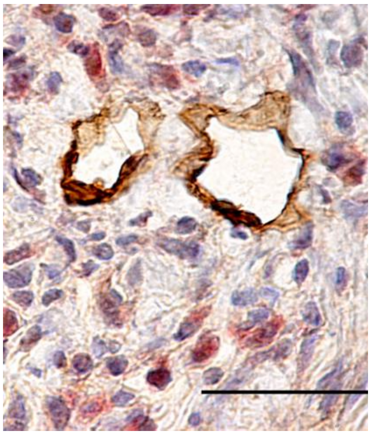

Supplemental Figure 2

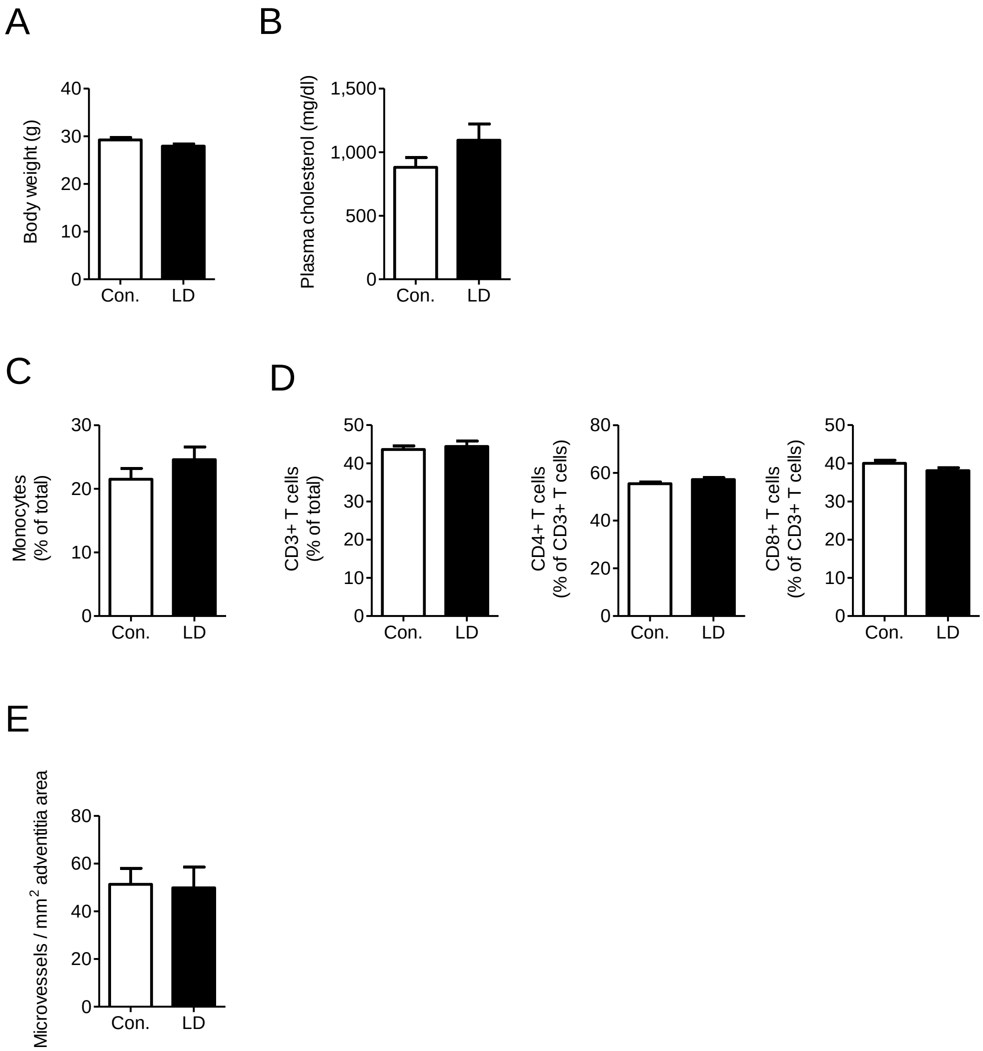

Supplemental Figure 3

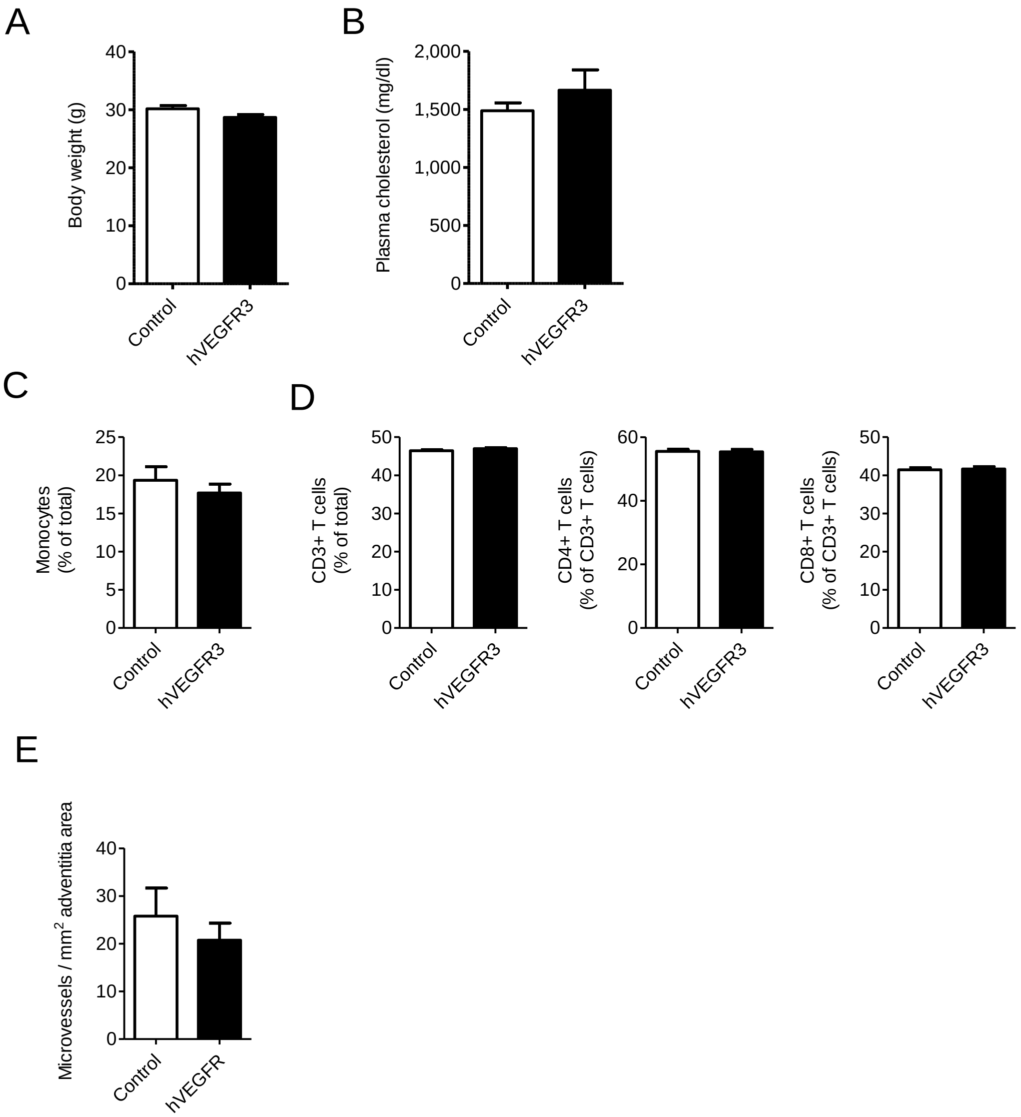

Supplemental Figure 4

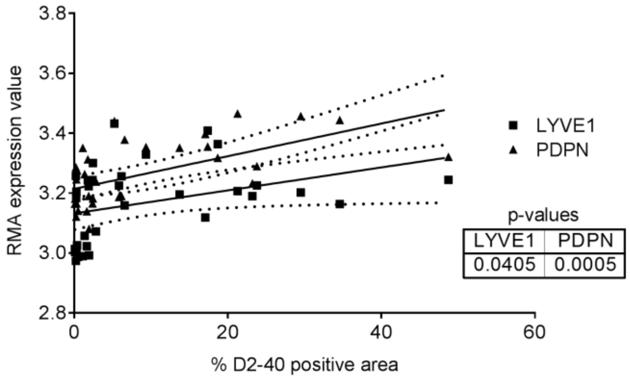

Supplemental Figure 1.

Lyve-1 (Brown)/Mac-3 (Red) double staining showing no overlap between Lyve-1 and Mac3 expression. Scale bar denotes 40µm.

Supplemental Figure 2.

General phenotype of mice after lymph node dissection and WTD, showing body weight (**A**), plasma cholesterol (**B**) and flow cytometry of blood monocytes (**C**) and T cells in peripheral lymph nodes (**D**). (**E**) Quantification of adventitial microvessels.

Supplemental Figure 3.

General phenotype of mice after AAV-hVEGFR3-Ig gene transfer and WTD, showing body weight (**A**), plasma cholesterol (**B**) and flow cytometry of blood monocytes (**C**) and T cells in peripheral lymph nodes (**D**). (**E**) Quantification of adventitial microvessels.

Supplemental Figure 4.

Correlation between podoplanin (PDPN) and Lyve-1 mRNA expression and relative D2-40^{+} LEC area (histology) in human endarterectomy plaque tissue.
